# Supplementary material for: The effect of common paralytic agents used for fluorescence imaging on redox tone and ATP levels in Caenorhabditis elegans
Source: PLoS One. 2024 Apr 26;19(4):e0292415. doi: 10.1371/journal.pone.0292415 (PMC11051652; doi:10.1371/journal.pone.0292415)
Supplement: S1 Table — Green shading indicates a statically significant p-value, while blue shading indicates a non-statistically significant p-value. (DOCX) [file pone.0292415.s001.docx]

|  | 1 min | 2 min | 3 min | 4 min | 5 min | 10 min | 15 min | 20 min | 25 min | 30 min |
| --- | --- | --- | --- | --- | --- | --- | --- | --- | --- | --- |
| 1 mM Levamisole | ns | ns | ns | ns | 0.0016 | <0.0001 | <0.0001 | <0.0001 | <0.0001 | <0.0001 |
| 3 mM Levamisole | ns | <0.0001 | <0.0001 | <0.0001 | <0.0001 | <0.0001 | <0.0001 | <0.0001 | <0.0001 | <0.0001 |
| 10 mM Azide | <0.0001 | <0.0001 | <0.0001 | <0.0001 | <0.0001 | <0.0001 | <0.0001 | <0.0001 | <0.0001 | <0.0001 |
| 100 mM Azide | <0.0001 | <0.0001 | <0.0001 | <0.0001 | <0.0001 | <0.0001 | <0.0001 | <0.0001 | <0.0001 | <0.0001 |
| 500 mM Azide | <0.0001 | <0.0001 | <0.0001 | <0.0001 | <0.0001 | <0.0001 | <0.0001 | <0.0001 | <0.0001 | <0.0001 |
| 0.5% 1P2P | ns | 0.0004 | <0.0001 | <0.0001 | <0.0001 | <0.0001 | <0.0001 | <0.0001 | <0.0001 | <0.0001 |
| 1% 1P2P | <0.0001 | <0.0001 | <0.0001 | <0.0001 | <0.0001 | <0.0001 | <0.0001 | <0.0001 | <0.0001 | <0.0001 |
| 100 mM 2,3-BDM | ns | ns | ns | ns | ns | <0.0001 | <0.0001 | <0.0001 | <0.0001 | <0.0001 |
| 300 mM 2,3-BDM | 0.0061 | <0.0001 | <0.0001 | <0.0001 | <0.0001 | <0.0001 | <0.0001 | <0.0001 | <0.0001 | <0.0001 |
| 4ºC Cold Shock | ns | ns | ns | ns | ns | ns | ns | ns | ns | ns |
