# Supplementary material for: The effect of common paralytic agents used for fluorescence imaging on redox tone and ATP levels in Caenorhabditis elegans
Source: PLoS One. 2024 Apr 26;19(4):e0292415. doi: 10.1371/journal.pone.0292415 (PMC11051652; doi:10.1371/journal.pone.0292415)
Supplement: S2 Table — P-values resulting from a two-way Kruskal Wallis test with Dunnet’s post-hoc are reported for percent recovery when compared to the control over time. Green shading indicates a statically significant p-value, while blue shading indicates a non-statistically significant p-value. (DOCX) [file pone.0292415.s002.docx]

|  | 15 min | 30 min | 45 min | 60 min | 75 min | 90 min | 105 min | 120 min |
| --- | --- | --- | --- | --- | --- | --- | --- | --- |
| 1 mM Levamisole | <0.0001 | <0.0001 | <0.0001 | <0.0001 | <0.0001 | <0.0001 | <0.0001 | <0.0001 |
| 3 mM Levamisole | <0.0001 | <0.0001 | <0.0001 | <0.0001 | <0.0001 | <0.0001 | <0.0001 | <0.0001 |
| 10 mM Azide | 0.0003 | 0.0147 | ns | ns | ns | ns | ns | ns |
| 100 mM Azide | <0.0001 | ns | ns | ns | ns | ns | ns | ns |
| 500 mM Azide | <0.0001 | <0.0001 | <0.0001 | <0.0001 | <0.0001 | <0.0001 | <0.0001 | <0.0001 |
| 0.5% 1P2P | ns | ns | ns | ns | ns | ns | ns | ns |
| 1% 1P2P | <0.0001 | <0.0001 | <0.0001 | <0.0001 | <0.0001 | <0.0001 | <0.0001 | <0.0001 |
| 100 mM 2,3-BDM | ns | ns | ns | ns | ns | ns | ns | ns |
| 300 mM 2,3-BDM | <0.0001 | <0.0001 | <0.0001 | <0.0001 | <0.0001 | <0.0001 | <0.0001 | <0.0001 |
| 4ºC Cold Shock | ns | ns | ns | ns | ns | ns | ns | ns |
