# Supplementary material for: The effect of common paralytic agents used for fluorescence imaging on redox tone and ATP levels in Caenorhabditis elegans
Source: PLoS One. 2024 Apr 26;19(4):e0292415. doi: 10.1371/journal.pone.0292415 (PMC11051652; doi:10.1371/journal.pone.0292415)
Supplement: Attachment — Submitted filename: Response to Reviewers.docx [file pone.0292415.s011.docx]

Dear PLoS One:

We greatly appreciate the reviewers’ comments and have made every effort to respond thoroughly to each comment. Substantial additions to the manuscript are that we quantified how the redox tone and relative ATP level change as a result of rotenone exposure would be altered by anesthetics that do and do not have an impact independently. Both experiments significantly strengthen our conclusion, and we thank reviewers for improving this manuscript. Our point-by-point responses are **in bold** below, and we have uploaded both tracked-change and clean versions of the manuscript.

Thank you,

Katherine Morton, Ashlyn Wahl, and Joel Meyer

**Point-by-point response:**

“For validation, this reviewer recommends the experiment where a representative chemical influencer to energetics or redox condition was administered to worms that received each one of ideal and inadequate anesthesia. Otherwise, the readers could think that significant differences should appear between the experimental and control groups, even under the worst anesthetic chemicals.”

**We have added two experiments to address this concern. As shown in Figures 7 and 8, we performed a rotenone exposure and examined how relative ATP and redox tone results compared when assessed with and without two anesthetics each. We demonstrate that even anesthetics not anticipated to have an impact would modulate the outcome of these experiments, with impacts ranging from alteration to the magnitude of difference between rotenone exposed and vehicle worms to unstable change over time in both treatment groups. While this does not comprehensively assess every anesthetic examined within the manuscript, we note that it is impossible to predict how each anesthetic would interact with all experimental designs and protocols. Rather, we aim to make the point that investigators must assess this outcome in their own experiments, and in the newly-added Figures 7 and 8 provide a proof of concept for what could happen.**

1. Lines 206-208. Please describe in detail. Fig 1C shows only one asterisk. Is this for the datum of 500 mM at 5 min or the data of total groups?

**Thank you for pointing out the lack of clarity. This asterisk was removed as it was from an earlier version of the statistics that did not include the cold shock. In the updated statistics, the point in question is not statistically significant. Due to the close overlap in groups within the same graph, we opted to include the statistical comparisons for these data within the supplement, as referenced in line 190.**

2. Line 243. “Fig 2A, 2B” should be corrected as “Fig 3B, 3C”, shouldn’t it?
3. Line 261. “Fig 4A” should be moved to just behind “(85.84%)”, shouldn’t it?
4. Line 263. “Fig 4B” should be moved as “Fig 4B, 4C” to just behind “3 minutes” on line 262, shouldn’t it?

**Thank you, these adjustments have been made as needed.**

5. Lines 262- 264. This reviewer cannot find the data to support this description in the manuscript.

**Experimental data and explanation has been added to support this information in Figure 4.**

6. Discussion. This reviewer is wondering if the authors briefly explain the pharmacological mechanisms of each anesthetic method from the viewpoints of total ATP, redox tone, and the paralytic effects.

**We have described what is known about the mechanisms of action of each of these with regard to paralysis and possible bioenergetic alterations in the Introduction. We have added a small section to the Discussion regarding possible mechanisms of altered redox state.**

The paper could be improved through addition of positive controls for the ATP and redox experiments, especially done in the same plate as levamisole where only minor changes were seen. Other ATP reducers such as oligomycin or orthogonal methods such as starvation could be used as positive controls. The authors mention the use of H2O2 as a control for redox experiments, but those data are not plotted anywhere and are missing values in the Supporting Information roGFP.csv file.

**We apologize for the lack of clarity for the use of positive controls. For the redox data for each paralytic, the positive H2O2 control has been added to the graph. For the ATP data, we respectfully suggest that the results in Figure 1 for sodium azide provide a sufficient positive control.**

While we understand the authors do not intend to be exhaustive and picked the concentrations used from the literature, it might be relatively easy and informative to do paralysis and recovery curves for multiple doses of each chemical (especially in cases where only two concentrations were tested). It would be interesting to see, for a given exposure time, if there is a certain threshold concentration (no worms paralyzed vs. 100% worms paralyzed) or if greater concentrations lead to greater percentages of paralyzed worms. It would also boost the utility of this paper as a resource for the worm community, for example, scientists looking for a faster than 15-minute half-maximal paralysis rate achieved with 100 mM 2,3 BDM but a greater recovery rate than that achieved with 300 mM 2,3-BDM.

**We have added doses such that all drugs have at least 3 doses assessed for paralysis and recovery. ATP and redox measurements were not always tested in as many concentrations. In particular, if lower concentrations resulted in large departure from the controls, it seemed unnecessary to test the higher concentrations.**

Similar to the point above, data in Figure 5 could be elevated if different time exposures to cold shock or if some combinations of cold shock and chemical agent (as the authors point out is a commonly used approach) were tested.

**While we recognize the desire to examine a wider utility of cold shock, it is experimentally used with a wider variety in methods than we can reasonably examine. Rather, we include it here to demonstrate that independently it does not induce impacts, but this would need to be verified in other experimental designs.**

Overall, greater detail in either the Methods or the Figure Legends of the data being presented would be good, for example, number of total independent replicates done, if one or more replicates are represented in the heat maps, the temperatures for ATP experiments given the temperature-dependence of the QUEEN-2m sensor. Some replicate information is presented in the Supporting Information, but would be more accessible to the reader if also stated in the text.

**We have added the number of biological replicates for each experiment and the temperature for Queen-2m measurements within the methods section.**
