# Supplementary material for: The effect of common paralytic agents used for fluorescence imaging on redox tone and ATP levels in Caenorhabditis elegans
Source: PLoS One. 2024 Apr 26;19(4):e0292415. doi: 10.1371/journal.pone.0292415 (PMC11051652; doi:10.1371/journal.pone.0292415)
Supplement: Attachment — Submitted filename: Resub 2_Response to Reviewer Comments_02.docx [file pone.0292415.s012.docx]

Dear PLoS One:

We greatly appreciate the reviewers’ comments and have made every effort to respond thoroughly to each comment. We have corrected previous grammatical and editing errors, and have added more clear explanations for our selection of doses in rotenone challenge experiments. Our point-by-point responses are **in bold** below, and we have uploaded both tracked-change and clean versions of the manuscript.

Thank you,

Katherine Morton, Ashlyn Wahl, and Joel Meyer

1. Line 210. What does this “d 100% by 30 minutesan” mean?

**This was an editing error and has been corrected.**
2. Line 213. The authors use “minutes” instead of “min” in other parts of this manuscript.

**We have corrected all use of “min” to “minutes” throughout the manuscript.**
3. Lines 325-346. This reviewer cannot understand why the authors chose the 3 mM levamisole despite the fact that 2 mM of levamisole worked efficiently enough and showed fewer side effects in Figure 2.

**Thank you for pointing out the lack of clarity for the choice of the 3 mM dose. A range of doses for levamisole are explored in the literature ranging up to 5 mM depending on the circumstances of the investigation. We selected 3 mM as a dose closer to those commonly used in experiments, and because the impact on relative muscle ATP levels is nearly identical to the 1 mM dose, demonstrating that 1 mM levamisole would likely still result in impacts. We note that while it may appear at first glance that 2 mM is a “Goldilocks” concentration given fast, 100% paralysis, we suspect it is not actually as perfect as it might appear. It would be very surprising if it were to work so significantly better than both 1 and 3 mM in terms of paralysis; we think a more conservative and likely true interpretation of the full dose-response data set is that all three concentrations behave similarly, and the apparently superior paralytic effect at 2 mM is a random result.**

**We emphasize that our goal is not to supply the perfect dose and anesthetic. Rather, we aim to demonstrate the potential impacts of a commonly used anesthetic and dose to underscore the requirement for researchers to validate their use of any chemical anesthetic in the future.**

**We have added sentences (lines 419-424) to clarify these points.**

Thank you for pointing out the lack of clarity for dose selection.
4. Figures 7 and 8. The authors added these new informative figures, considering the previous comments. Unfortunately, this reviewer could find no description of how the authors calculated the fold changes of the Y-axis in panels B of both figures, reading the figure legend or “Materials and methods.”

**Thank you for pointing out this lack of description. We have added the method used to calculate fold change into the figure description of both Figures 7 and 8.**

5. Line 394. Please insert a space between “(12)” and “and.”

**Thank you, we have corrected this.**
6. Lines 453-457. This reviewer admires the scientific manner of the authors and the modest description. However, this reviewer still needs to wonder why the authors hesitate to describe the conclusion. The data in Figures 2 and 6 likely indicate that 1- or 2-mM levamisole would be the first choice, subject to the authors providing the revised Figure 7, where 1- or 2-mM levamisole should be examined. Although 100 mM BDM looked like another suitable method in Figures 4 and 6, the new Figure 8 did not accept it. Cold shock looks safe and ideal but needs more time to get even the incomplete paralysis.

**Thank you, we deeply appreciate your compliment. We believe we have addressed the concerns regarding levamisole above. We have also added two sentences (lines 464-466 and 472-474) to more clearly describe our conclusions.**
